# Informer-Based Temperature Prediction Using Observed and Numerical Weather Prediction Data

**DOI:** 10.3390/s23167047

**Published:** 2023-08-09

**Authors:** Jimin Jun, Hong Kook Kim

**Affiliations:** 1 School of Electrical Engineering and Computer Science, Gwangju Institute of Science and Technology, Gwangju 61005, Republic of Korea; jiminbot20@gm.gist.ac.kr; 2 AI Graduate School, Gwangju Institute of Science and Technology, Gwangju 61005, Republic of Korea

**Keywords:** temperature prediction, informer-based model, model fusion, numerical weather prediction (NWP), local data assimilation and prediction system (LDAPS)

## Abstract

This paper proposes an Informer-based temperature prediction model to leverage data from an automatic weather station (AWS) and a local data assimilation and prediction system (LDAPS), where the Informer as a variant of a Transformer was developed to better deal with time series data. Recently, deep-learning-based temperature prediction models have been proposed, demonstrating successful performances, such as conventional neural network (CNN)-based models, bi-directional long short-term memory (BLSTM)-based models, and a combination of both neural networks, CNN–BLSTM. However, these models have encountered issues due to the lack of time data integration during the training phase, which also lead to the persistence of a long-term dependency problem in the LSTM models. These limitations have culminated in a performance deterioration when the prediction time length was extended. To overcome these issues, the proposed model first incorporates time-periodic information into the learning process by generating time-periodic information and inputting it into the model. Second, the proposed model replaces the LSTM with an Informer as an alternative to mitigating the long-term dependency problem. Third, a series of fusion operations between AWS and LDAPS data are executed to examine the effect of each dataset on the temperature prediction performance. The performance of the proposed temperature prediction model is evaluated via objective measures, including the root-mean-square error (RMSE) and mean absolute error (MAE) over different timeframes, ranging from 6 to 336 h. The experiments showed that the proposed model relatively reduced the average RMSE and MAE by 0.25 °C and 0.203 °C, respectively, compared with the results of the CNN–BLSTM-based model.

## 1. Introduction

The prediction of future atmospheric temperature is referred to as temperature prediction, which is one part of a weather forecasting system. The importance of temperature predictions is increasing with the acceleration of climate change. The conventional methodology used for weather forecasting, including temperature prediction, largely relies on numerical weather prediction (NWP) models [1]. These models entail a complex suite of procedures for predicting the future state of the atmosphere by solving intricate physics and dynamics equations that encapsulate atmospheric motion and the changes within it.

Despite the good performances of NWP models, they exhibit a critical shortcoming: their accuracy diminishes with the acceleration of global warming and the resulting climate change [2]. As a response, the authors of many studies have examined the use of deep-learning-based predictions and forecasts for various meteorological variables [3,4,5,6]. Recurrent neural networks (RNNs), with their ability to predict current or future states based on past information, have been utilized in numerous studies to predict future time step variables [7,8,9,10,11]. However, with increasing network depths, RNNs often experience gradient vanishing during the learning process. Furthermore, a long-term dependency issue arises when the gap between the perspectives expands, leading to a decrease in the influence of distant past information on the current time step.

To address this problem, Transformer models have emerged as potential alternatives to tackle the long-term dependency issue [12]. Designed to represent the relationships across different time steps [13], Transformer models enhance the capacity to predict long sequences. Having a long-range alignment capability and the capacity to efficiently process long sequences as both inputs and outputs are crucial requirements for this task. In comparison with other network structures, Transformers demonstrate superior long-range alignment capabilities. They are considered to be apt for predicting meteorological variables, including temperature, due to their ability to bypass the long dependency problem in long-term forecasting. However, the self-attention mechanism of Transformers does not fulfill the requirements of effective operations under lengthy sequence conditions.

Numerous models modifying the Transformer architecture have been scrutinized in order to address the aforementioned issues of long-term dependency and a computational burden. Among these, the Informer model has successfully rectified these problems and demonstrated an excellent performance in time series prediction [12]. The proposed solution was the ProbSparse method. This method enhances the computational and memory efficiencies using a self-attention mechanism, thereby enabling the prediction of extended time series sequences through a single forward step facilitated by a generative-style decoder. Given these strengths, we introduce a novel methodology for temperature prediction, employing the Informer model.

In this paper, we propose a method to predict the long short-term temperature via the fusion of features of the time series data extracted using the Informer-based network, and the features of channel-wise NWP data, extracted using the convolutional neural network–bidirectional long short-term memory (CNN–BLSTM) method. Additionally, we explore a multimodal learning approach that combines domain data from various dimensions. In the context of this study, our aim was to predict the temperature, a one-dimensional time series variety of data, by concurrently analyzing observational data and NWP data, constituting time series data and image data, respectively. We evaluated the efficacy of this fusion by determining how well the two vectors, extracted from the respective features, are integrated within the learning process. Our contributions are as follows: (1) this is a new approach to temperature prediction with the Informer using one-dimensional observed data; (2) this is a multimodal study combining one-dimensional observed data and two-dimensional NWP image data; and (3) this research focuses on fusioning using various multimodal learning approaches.

The remainder of this paper is organized as follows. Section 2 describes the traditional weather forecast models used in meteorology and reviews various examples of deep learning techniques applied for time series prediction and weather variable prediction. Section 3 describes the types of data used, including temporal and spatial information, and explains the previous state-of-the-art model used for temperature predictions. Section 4 proposes an Informer-based temperature prediction model with data preprocessing and the fusioning of AWS and LSAPS data. Section 5 compares and discusses the performance of the proposed model with that of the previous model. Section 6 summarizes and concludes the paper.

## 2. Deep-Learning-Based Temperature Prediction Methods

Conventional weather forecasting methodologies that employ NWP models [14,15] apply a sequence of equations grounded in a regulated physical setting to the dynamical evolution of atmospheric conditions within a small hexahedral volume [16]. This can be extrapolated to a larger air mass to anticipate the air circulation and meteorological conditions [17]. Since the 1980s, NWP models have been substantially advanced. This can be attributed to their increased computational power, superior modeling techniques, and enhanced data assimilation accuracy [3]. However, NWP exhibits certain limitations, such as ambiguities in the initial state of the model and boundary conditions [18], simplifications in the surface attributes and their influence on the model’s output [19], and the model’s structural design and its approximation to reality [20].

Lately, deep-learning-based models have surfaced as alternative solutions to the challenges associated with NWP. These models demonstrate the capability to decipher complex, non-linear relationships inherent in data, a quality beneficial for weather forecasting. Traditional numerical weather prediction models, such as those based on Navier–Stokes equations [21], are reliant on mathematical equations that delineate the physical processes dictating weather systems. Although NWP models can yield accurate forecasts under certain circumstances, they may fail to capture specific weather patterns or predict extreme events.

In contrast, deep learning models possess the capacity to identify patterns in vast datasets, such as meteorological observations and prior forecasts. This enables them to generate predictions that are better those produced by traditional numerical models, particularly for short-term forecasting. Moreover, deep learning models are capable of automated feature extraction. This enables the automatic identification of significant features in the input data, thereby enhancing the forecast accuracy. These models can also process copious amounts of observational data and satellite imagery, which is useful for generating the initial conditions for numerical models. This process, known as data assimilation, can augment the precision of forecasts produced by NWP models.

The kind of method used depends on the type of data under investigation. Research has been conducted using CNN-based models for two-dimensional images, and RNN-based models have been used for one-dimensional time series data. CNN-based models can effectively represent the spatial aspects of weather phenomena [22,23,24,25]. Currently, satellite imagery or NWP outputs that encapsulate spatial attributes are employed as inputs. Some studies have predicted the likelihood of precipitation using radar and satellite data [26,27], as well as research forecasting hail by taking NWP variables such as temperature, dew point, and wind as inputs [28]. Furthermore, it was demonstrated that a CNN-based generative model can provide an accurate short-term precipitation probability prediction, thus addressing the issue of inaccurate heavy rain forecasts with lengthy lead times [29].

Time series one-dimensional data are the focus of RNN-based prediction. Numerous studies have been performed for the purpose of using RNN structures to forecast meteorological variables [7,8,9,10,11]. Recent studies have also incorporated both spatial and temporal data into models, facilitating the usage of more diverse and abundant meteorological data as inputs [24,30,31,32]. LSTM can be employed to extract temporal features from the input data; CNNs can be used to extract spatial information. The key to this type of model lies in how to effectively combine spatial and temporal data for research purposes. Consequently, by experimenting with various fusion models of spatial and temporal features, an optimal fusion model is developed in this study.

As mentioned above, RNN models, particularly LSTM, are widely used to handle time series data. However, recent studies on time series prediction have begun to adopt Transformer models [12,33,34]. One of the key advantages of using Transformer models over LSTM models lies in their capacity to effectively manage extremely long data sequences. LSTM models are engineered to manage data sequences in which the initial information is vital for comprehending the final information; however, they can struggle with sequences that are overly lengthy or display intricate dependencies between elements [9]. In contrast, the Transformer model uses self-attention mechanisms to assess the significance of distinct elements in the input sequence, enabling it to manage extremely long sequences and effectively capture intricate dependencies between elements.

Another advantage of the Transformer model is its proficiency in performing parallel computations, and it can outpace the LSTM models. This is attributed to the self-attention mechanism, which enables the models to make predictions for each sequence element independently of the others, whereas LSTM models necessitate elements to be processed in a specific order. Furthermore, Transformer models are more adaptable in terms of handling multiple inputs simultaneously, whereas LSTM models are optimized for applications to sequential data. Transformer models can, thus, exhibit greater flexibility in modeling different types of inputs. In conclusion, Transformer models outperform LSTM in managing long sequences and complex dependencies and are also computationally more efficient. Moreover, among the different alternatives of Transformer models, the Informer model is known to be suitable for time series prediction [12]. Consequently, in this study, an Informer-based model was used instead of an LSTM for feature extraction from temporal data.

## 3. Datasets and Conventional Methods

### 3.1. Datasets

In this study, two types of weather observation data produced and distributed by the Korea Meteorological Administration (KMA) were used: observation data and numerical forecast models. Observation data are data directly observed from a terrestrial environment, marine environment, or local weather at a high altitude; these data include AWS, AAOS, AMOS, ASOS, and Rawinsonde data. Only AWS data were considered in this study. AWS data are ground observation data produced using an automatic weather system (AWS) operated by KMA, and they are observed at approximately 510 points across the country. The meteorological variables of AWS include temperature, precipitation, wind, humidity, and barometric pressure. Here, we used five types of variables—temperature, accumulated precipitation, and average wind direction, wind speed, and humidity—obtained from the station in Dobong-gu, Seoul (area code 406). Barometric data were excluded because they do not deviate significantly from 1000 hPa at any point in time as they predict the layer temperature. The goal was to predict the temperature in units of time; therefore, the accumulated precipitation over one hour was selected so that weather information could be obtained for one hour. Notably, air quality data are known to be highly correlated with temperature [35], but these data were not used in this study because the AWS does not include them.

Table 1 describes the details of three different models, namely, the global data assimilation and prediction system (GDAPS), the regional data assimilation and prediction system (RDAPS), and the local data assimilation and prediction system (LDAPS). These three models are built on the unified model (UM) made by the British Met Office. They are representative numerical models used by the Korea Meteorological Administration (KMA). Each model has a distinct prediction time, horizontal grid size, and spacing. In this study, LDAPS was chosen because it has the highest spatial and temporal resolutions among the provided NWP data. If the spatial resolution is high, more accurate information can be applied using the model when extracting spatial features through CNNs. The higher the time resolution value is, the shorter the forecast period will be. In this experiment, training data were input in units of 1 h. However, NWP data are not recorded every hour; thus, they were generated in units of 1 h via linear interpolation. At this time, if the temporal resolution increases, the error between the actual value and the generated interpolation data decreases. The spatial resolution of LDAPS is 1.5 km, its forecast cycle is 3 h, and it is composed of 70 layers, reaching up to about 80 km vertically.

The output data of the NWP are provided in three layers: a model layer, an isobaric layer, and a single layer. The model layer refers to the vertical layer in NWP, and the isobaric layer has the characteristic of expressing the atmospheric state in three dimensions by interpolating the meteorological elements calculated on the model layer into standard isobaric values. In this study, we judged it to be appropriate to use the same 10,000 Pa layer by applying it in combination with the data observed on the ground. Among the variables of the isobaric layer, a temperature of 10,000 Pa, the u and v components of wind, and relative humidity were used; among the single-layer variables, large-scale precipitation was used as the input data.

In this experiment, data collected over approximately 3 years and 3 months were used. The period lasted from 10 September 2012 to 31 December 2015. In the total dataset, 80% of the values were used as training data, 10% were used as validation data, and 10% were used as test data. The specific period of each dataset was as follows: training data comprised data collected from 00:00 on 10 September 2012 to 18:00 on 4 September 2014; validation data comprised data from 19:00 on 4 September 2014 to 08:00 on 4 May 2015; and test data comprised data from 09:00 on 4 May 2015 to 23:00 on 31 December 2015.

### 3.2. Conventional Methods

**Figure 1:**
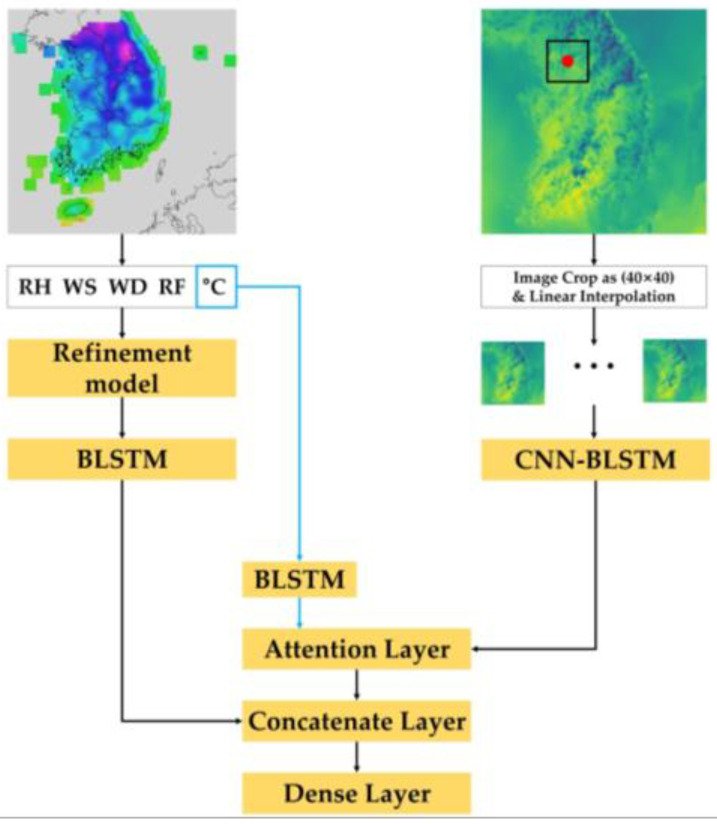
Block diagram of the prior temperature prediction model [32]. This model extracts features from observed data and 2D NWP data separately. It uses attention mechanisms and a concatenate layer to connect these features, and then predicts temperature using subsequent dense layers.

A prior study [32] proposed a model that learns through deep learning using observation data and NWP data simultaneously. Therefore, it was used as a baseline model in this study, and the model is described in detail in this section. Figure 1 shows the architecture of the previously proposed model [32]. In this study, the authors developed a temperature prediction model based on deep neural networks that makes use of the observed time series weather data and RDAPS image data, which are listed along the time axis as 1D and 2D data, respectively. This prediction model performs three functions: feature representation, information fusion, and prediction. For the feature representation, two distinct neural networks were employed to integrate the diverse input data sources. The observed time series data and the RDAPS image data were processed using a BLSTM neural network and a CNN–BLSTM neural network.

Hence, to address these issues, we substituted LSTM with the Informer model, which demonstrated robustness against long-term dependency problems. Furthermore, we introduce a method for combining diverse representations of observational data and numerical weather prediction image data. During the learning process of deep neural networks, the vanishing gradient problem is encountered, which signifies the loss of the gradient. If the interval between time points is expanded, a long-term dependency issue arises, where the influence of information from a distant past point is attenuated in relation to the current time point. Thus, as a solution to these problems, we propose replacing LSTM with the Informer model, which is resilient to long-term dependency issues. Additionally, we propose a strategy for integrating various forms of observational data and numerical weather prediction image data in a comprehensive manner.

## 4. Proposed Temperature Prediction Model

**Figure 2:**
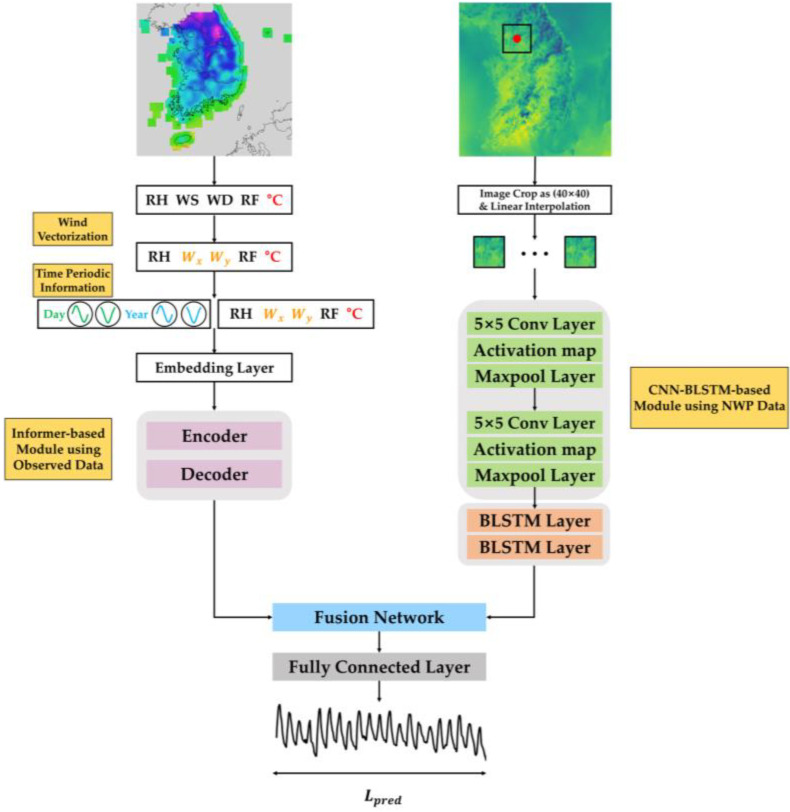
Block diagram of the proposed methods. Generated time-periodic information is appended to the observed data along the channel axis after applying wind vectorization. The 2D NWP images are processed via the use of a CNN–BLSTM-based module to extract relevant features. The Informer model utilizes observation data as inputs for both the encoder and decoder. Various fusion techniques are employed to incorporate the features from the 2D NWP image into the Informer model. The fused features are then propagated through the model and, ultimately, the temperature is predicted using a fully connected layer.

Figure 2 shows the overall architecture of our proposed model. In short, this model takes two types of data as inputs of the model, i.e., AWS and LDAPS, and inputs these data through data processing. AWS undergoes the process of refining missing values and vectorizing scalar wind-related variables. In addition, time-periodic information is added to reflect the periodicity of the weather. LDAPS compensates for missing values through interpolation and performs hourly refining and image cropping. After data pre-processing, each data feature is extracted through the Informer and CNN–BLSTM structures. Subsequently, in the fusion network, various fusion methods are used to convert two features into a single vector. Ultimately, the final temperature is predicted through a fully connected layer.

### 4.1. Pre-Processing

Meteorological data might be missed due to various reasons, such as inspections or breakdowns. This is a concern when one is directly applying model learning. In addition, it is necessary to transform and refine the data in a more suitable way for model training. Therefore, various pre-processing techniques are applied. Data normalization is also commonly applied to guarantee correct learning by the model and fast convergence. For AWS, two pre-processing techniques are applied: refining the missing time steps and wind variable vectorization. The approach outlined in [36] is used to refine the missing data from AWS. Next, wind variable vectorization is applied. AWS data provide wind speed and wind direction. Converting wind direction and wind speed into wind vectors is expected to render the model easier to interpret. Therefore, wind direction and wind speed are vectorized using the following equations:

(1) $$w_{x}=w_{v}\cdot \cos w_{d}\;\mathrm{and}\;w_{y}=w_{v}\cdot \cos w_{d}$$

where $w_{v}$ and $w_{d}$ are the wind speed and direction, respectively, and $w_{x}$ and $w_{y}$ are the *x* and *y* components of wind, respectively.

For NWP, two preprocessing techniques are applied: refining missing data and image cropping. In NWP, the time step is not missing on the time axis, but it is irregularly missing at specific two-dimensional coordinates. By referring to normal data around the missing values, the image is interpolated by filling the missing values with the average value of a 3 × 3 filter. NWP has no missing values on the time axis. However, NWP produces results four times a day at 6 h intervals, while LDAPS produces results eight times a day at 3 h intervals. Given that the time resolution of AWS is 1 h, it is necessary to set the NWP data at 1 h intervals when inputting the model values.

**Figure 3:**
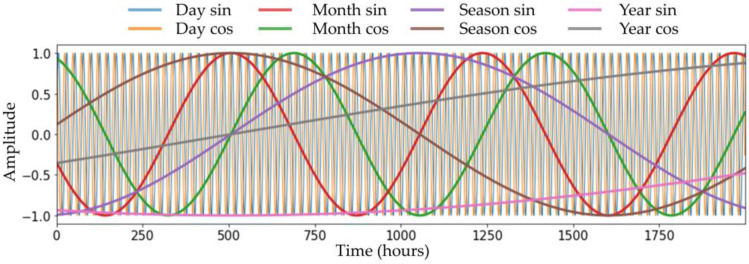
Plot of different time-periodic information. This was plotted by generating time-periodic information with different periods. There was a total of four periods applied: day, month (30 days), season (90 days), and year (365 days). Eight types of time-periodic information were created by generating sin and cos signals for each period. For example, Day sin is a sin signal, with 24 h as a single cycle period.

Next, it is necessary to crop the NWP to fit the model. The NWP calculates data for a very large area centered on the Korean Peninsula. Therefore, the image is cropped in a manner that was heuristically suitable for the purpose of predicting the temperature of the ground observatory located in Seoul. NWP is cut to a size of 40 × 40 to cover the South Korean area. Time-periodic information is an arbitrarily generated signal with a constant period, as shown in Figure 3. It is common knowledge that temperature has a periodic characteristic due to the periodic revolution and rotation of the Earth. Thus, four different periods—day, month, season (3 months), and year—were modeled in this study to provide information on the seasonality and repetition of weather. We chose these four periods based on heuristic knowledge that the period of Earth’s rotation is one day, and the period of Earth’s revolution is one year. The month and season were set as such by considering the change in seasons as a period according to the convention. Specifically, a day was set to 24 h, a month was set to 30 × 24 h, a season was set to 3 × 30 × 24 h, and a year was set as an approximate value of 365 days and 6 h.

### 4.2. Informer-Based Temperature Prediction Using Observed Data

**Figure 4:**
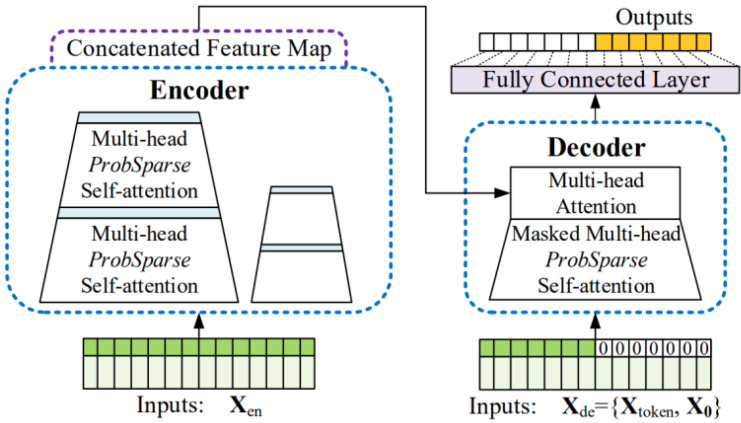
Architecture of the conventional Informer that was used for the proposed temperature prediction models (Reprinted/adapted with permission from Ref. [12]. Copyright 2023, AAAI Press).

This section describes a model that uses observation data as inputs using the Informer, as shown in Figure 4. Prior to this, the detailed structure and methodology of Informer is described. The Informer is an encoder–decoder structure and decides a fully connected final layer. In general, the encoder–decoder structure receives the input through an encoder, generates a hidden representation as the encoder output, and then sequentially receives the predicted output from the decoder and performs decoding. However, the Informer predicts within a single forward step, rather than performing sequential prediction. The input sequence length of the Informer encoder is referred to as $L_{seq}$, its start token length is referred to as $L_{label}$, and its prediction sequence length is referred to as $L_{pred}$. The input sequence length of the Informer decoder is the sum of $L_{label}$ and $L_{pred}$.

Before forwarding the input to the encoder and decoder, embedding was performed for the uniform input representation. The pointwise self-attention technique used by the vanilla Transformer [8] employs time stamps to provide the local positional context. However, the capacity to represent long-range independence in the long-range dependency problem necessitates the use of global data such as hierarchical time stamps (week, month, and year) and agnostic time stamps (holidays and events).

The encoder of the Informer is composed of an embedding layer and stacks of attention and convolution blocks. After the use of ProbSparse self-attention, distilling is performed through convolution and max pooling. This is performed to construct information for transmission to the next layer by extracting only the important information from the attention output. The decoder employs masked ProbSparse self-attention, as with the encoder and multi head attention. Encoder–decoder attention uses the same attention as the vanilla Transformer. Then, the decoder output is fed into a fully connected layer to construct the prediction output, $L_{pred}.$ To train the Informer, the MSE loss, which is the difference between the target value and the prediction value, is used as the loss. ProbSparse attention, designed to reduce the amount of computation, is applied. Its application starts under the premise that it is inefficient to create all the dot products between the query and key. New selective counting strategies are proposed that exclude those techniques that significantly affect the dot products between the query and key.

### 4.3. Informer Fusion with CNN–BLSTM Using NWP

**Figure 5:**
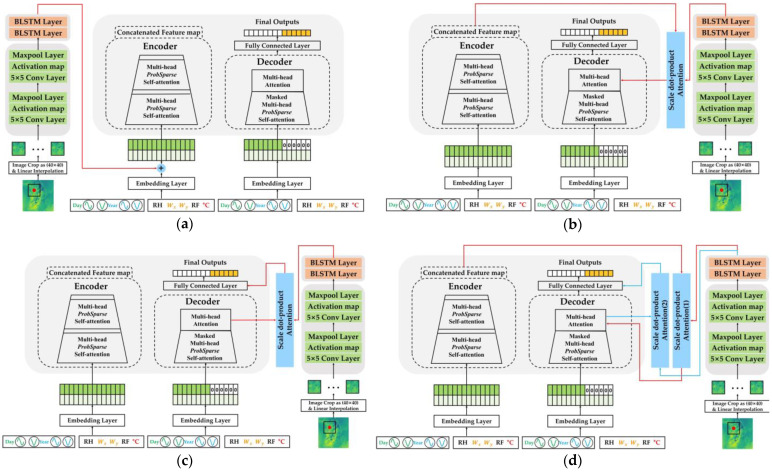
Architectures of various ways of fusioning the Informer-based model using observed data and CNN–BLSTM-based model using NWP data: (**a**) Informer addition model; (**b**) Informer encoder fusion model; (**c**) Informer decoder fusion model; and (**d**) Informer encoder–decoder fusion model.

In this section, each module is described before fusion. Figure 5 shows four different fusion models. To evaluate the objective performance of the Informer, only AWS data, which are one-dimensional time series values, were applied and tested. The Informer was trained by setting the temperature as a target and using five types of meteorological variables: air temperature, the x and y components of the wind vector, precipitation, and relative humidity. The length of the encoder input is $L_{seq}$. The length of the decoder input is the sum of $L_{label}$ and $L_{pred}$. $L_{label}$ represents data observed during model training, while $L_{pred}$ is the unseen data padded with zeros for the length of the target prediction.

First, local embedding was applied to the meteorological variables, and global embedding was applied by setting the time information as features of the day, month, day of the week, and time. Then, the added embedding vector was given as the input to the encoder and decoder. After conducting multi-head ProbSparse self-attention in each module, the attention focused on the output vector, the one-dimensional vector that had passed through the fully connected layer was indexed from the back as much as the prediction time, and finally, a decision was made. The following is an explanation of what occurs when NWP inputs are stacked as channels. The module that extracts the features of LDAPS stacks the image field data of the added NWP in the channel direction and uses it as an input. Then, when two convolutional blocks consisting of one convolution layer, rectified linear unit (ReLU) activation, and a max pooling layer are passed, a feature map of size (6, 6, 256) is obtained. At this time, the kernel size of the first convolution layer is (5 × 5), the channel size is 64, the kernel size of the second convolution layer is (7 × 7), and the channel size is 256. A flattened vector is then created, and this process is stacked using the $L_{seq}$ set for each time step to finally generate a feature representation of size ($L_{seq}$, 9216).

In order to improve the performance, we propose a method for fusing the structures of CNN–BLSTM using NWP and Informer using AWS data in various ways. We propose four distinct types of Informer and CNN–BLSTM fusion. The CNN–BLSTM module has the same structure as that seen in the conventional model, and it is a structure that predicts the temperature through a fully connected layer after feature extraction via CNN–BLSTM using only NWP. Fusion processes were introduced with the intention that they would integrate the relationship between the observed data and the information of the NWP image and transmit it to the subsequent layer.

We aimed to find the most effective fusion method by conducting experiments with various techniques, leveraging features extracted via the Informer from the observation data and features taken from the NWP via augmented channels. In the Informer addition model, the Informer encoder’s embedded input and the CNN–BLSTM’s feature extraction output vectors are added together. This sum is then used as the Informer encoder’s input. The Informer encoder fusion model utilizes scaled dot product attention between the CNN–BLSTM’s feature extraction output vector and the Informer encoder’s output. The scaled dot-product method scores the correlation between distinct time steps using the dot products of two different vectors, thereby enabling effective comprehension.

The Informer decoder fusion model applies the scaled dot product attention between the CNN–BLSTM’s feature extraction output vector and the Informer decoder’s output. The Informer encoder–decoder fusion model utilizes scaled dot-product attention between the CNN–BLSTM’s feature extraction output vector and the Informer encoder’s output. Subsequently, the context vector obtained from the previous attention is derived from the masked multi-head ProbSparse attention vector and conventional attention in the decoder module. The resulting vector obtained from the feature extraction output vector of CNN–BLSTM via scaled dot product attention is then processed through a fully connected layer to predict subsequent time points.

## 5. Experiments and Discussion

### 5.1. Experimental Setup

In every experimental case, the cross-entropy loss between the label and the prediction was selected as a cost function. The weights were initialized via Xavier initialization [37], while all the biases were initialized as zero. An adaptive moment estimation (Adam) optimizer [38] was utilized for the backpropagation algorithm. The batch size was set to 64. The epoch was set to 200, and the early stopping method was applied to stop the validation loss if it did not decrease to the same degree as that of patience. At this time, patience was set to 10, and the learning rate was set to 0.0001. The performance of each of the temperature prediction models was evaluated using:

(2) $$\mathrm{RMSE}=\sqrt{\frac{1}{N}\overset{N}{\underset{t=1}{\sum }}{\left(v_{t,\;pred}-v_{t,obs}\right)}^{2}}$$

(3) $$\mathrm{MAE}=\frac{1}{N}\overset{N}{\underset{t=1}{\sum }}\left|v_{t,pred}-v_{v,obs}\right|$$

where $v_{t,pred}$ denotes a predicted vector, whose length is $L_{pred}$ and which starts from *t*, $v_{t,target}$ denotes a target vector with length $L_{pred}$ and starting from *t*, and N is the number of elements in the vectors.

### 5.2. Performance Evaluation

Table 2 summarizes an experiment that was conducted in order to determine whether the Informer performed better than BLSTM, which is the most used model among the models that input only observation data. At this time, the BLSTM model consisted of two BLSTM layers with 256 hidden nodes and a fully connected layer. $L_{seq}$, $L_{label}$, and $L_{pred}$ were set to (24, 12, 6), (96, 96, 12), (168, 168, 24), (336, 168, 72), (168, 168, 168), and (168, 168, 336) for 6, 12, 24, 72, 168, and 336 h future temperature predictions, respectively. The inputs of both the BLSTM-based and Informer-based prediction models were the same for five observation data variables.

As shown in the table, the Informer-based model achieved lower RMSE and MAE values for all the prediction times in comparison with those of the BLSTM-based model. Compared with the BLSTM-based model, the Informer-based model reduced the RMSE and MAE by 0.76 °C and 0.55 °C, respectively, when 6 h prediction was performed. Even though the prediction time was increased to 336 h, the Informer-based model also reduced the RMSE and MAE by 0.37 and 0.25, respectively, compared with the BLSTM-based model.

Table 3 summarizes the results of applying the Informer in various ways. In this experiment, wind vectorization and time-periodic information were consistently applied to AWS data. In practice, this meant that temperature, accumulated precipitation for 1 h, the x component of the wind vector, the y component of the wind vector, relative humidity, and four types of time-periodic information were applied to the AWS data. LDAPS was applied to the NWP data. For the training settings, the epoch was set to 500, early stopping was applied with a patience of 20, and the learning rate was set to 0.0001. As detailed in the table, the models were trained via a cosine annealing warm restarts scheduler [39]. To obtain detailed parameters of the scheduler, we set the maximum learning rate to 0.0001, adopted an initial cycle value that was four times larger than the length of the train loader, reached the maximum learning rate in 10 epochs, and set it to be 0.5 times larger than the maximum learning rate in subsequent cycles. The experiment was conducted with the values of $L_{seq}$, $L_{label}$, and $L_{pred},$ which were all set to the same value as the predicted time.

As shown in the table, the different fusioning of the Informer-based model provided different performance results. In fact, the performance of the Informer encoder fusion model showed the lowest RMSE and MAE values when the prediction times were greater than or equal to 24 h, while that of the Informer encoder–decoder fusion model was the best when the prediction times were smaller than 24 h. However, the performance results of the Informer embedding addition model and Informer decoder fusion model were worse than those of the Informer encoder fusion model and Informer encoder–decoder fusion model. The reason why this result was obtained is due to the fusioning method used on the LDAPS data and AWS data. In other words, as shown in Figure 5a, LDAPS image data were directly fused into the embeddings of AWS data. Thus, the feature representation of LDAPS image data was different from that of AWS data regarding vector embedding. Similarly, the Informer decoder fusion model combined the decoded AWS data and LADPS embedding vector, as shown in Figure 5b. Consequently, fusioning methods should combine AWS and LDAPS data in a level of embedding vectors, as shown in Figure 5c,d.

Next, we compared the performance of the Informer fusion model with that of the CNN–BLSTM fusion model. The Informer encoder–decoder fusion model achieved RMSE and MAE reductions of 0.07 °C and 0.05 °C at the 6 h prediction time, respectively, which were compared with those of the CNN–BLSTM fusion model. When the models were simulated at the 12 h prediction time, the Informer encoder–decoder fusion model reduced the RMSE and MAE by 0.47 °C and 0.34 °C, respectively. When the prediction time increased to 24 h, the Informer encoder–decoder fusion model still improved more than the temperature prediction model and the CNN–BLSTM fusion model, but the improvement was marginal. However, the Informer encoder fusion model was employed for the temperature prediction, and it could reduce the RMSE and MAE by 0.25 °C and 0.19 °C, respectively. The relative reductions of the RMSE and MAE were also observed even when the prediction time was 336 h, where the Informer encoder fusion model was the best among all the fusion models; it lowered the RMSE and MAE by 0.29 °C and 0.20 °C, respectively, which were compared with those of the CNN–BLSTM fusion model.

**Figure 6:**
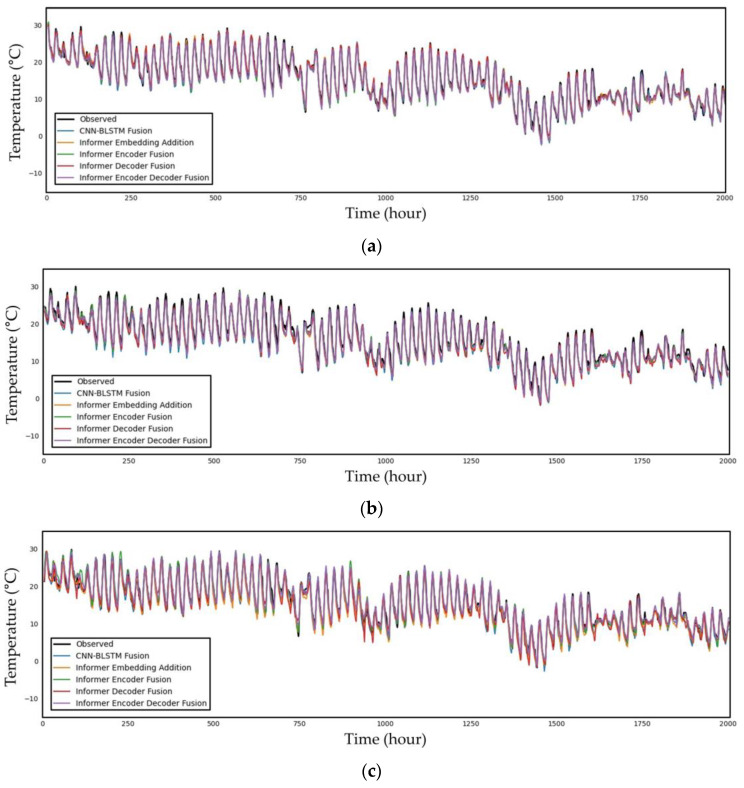

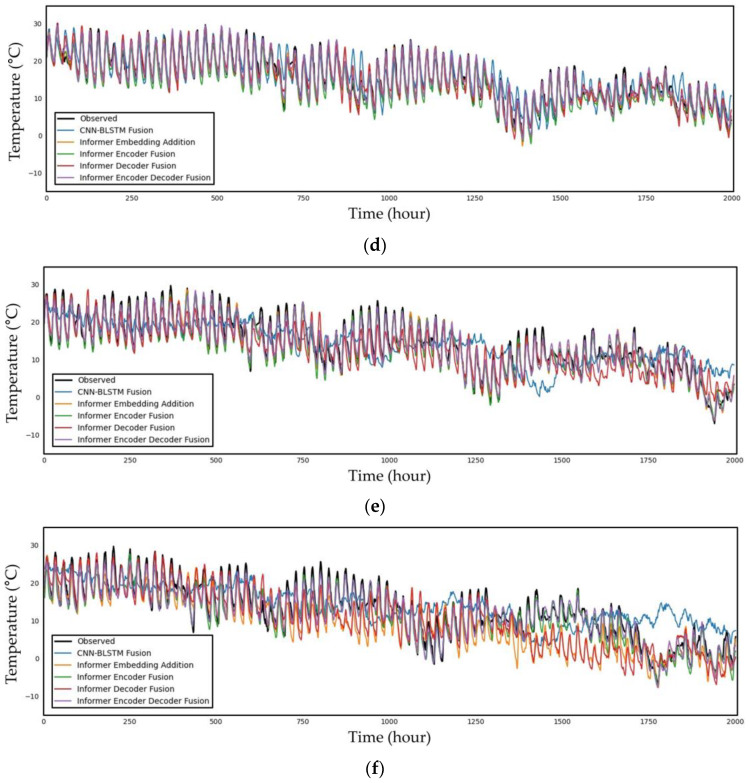
Time series plot of the observed and predicted temperature data from 09:00 on 4 May 2015 to 23:00 on 23 December 2015 for the (**a**) 6 h, (**b**) 12 h, (**c**) 24 h, (**d**) 72 h, (**e**) 168 h, and (**f**) 336 h predictions.

Finally, Figure 6 illustrates a time series plot of the observed data and predicted temperature data from 09:00 on 4 May 2015 to 23:00 on 23 December 2015 for the 6, 12, 24, 72, 168, and 336 h future predictions. As shown in the figure, the shorter the forecast period is, the better the prediction graphs reflect the trend in the observed data graph. There are more portions where the graph does not match as the prediction time grows, and in the case of the 336 h prediction, it is evident that the predicted graph is drawn somewhat differently from the graph of the observed data in a significant number of sections. For the 6 to 24 h predictions, which are relatively short-term graphs in the figure, all the models appear to closely resemble the observed data graph, making it challenging to determine which model is the best with a human eye. This contrasts with the prior model, the CNN–BLSTM fusion model.

However, we can clearly observe that, as the prediction hour increases from 72 h to 336 h, the difference between the predicted values from models and the observed values becomes more pronounced. Although it closely resembles the periodic pattern in the 72 h forecast graph, there is a difference in the magnitude. The time point between 1250 and 1500 h in the 168 h prediction can be seen to significantly diverge from the prediction; this is the juncture where the disparity across models is most pronounced. The Informer fusion models are proven to reflect the periodicity well; although, there are changes in the degree. However, the CNN–BLSTM fusion model shown in blue in the 168 h prediction graph fails to follow both the periodicity and the trend lines. When compared with the observed data, the degree of error increases significantly at around 336 h. In the case of the CNN–BLSTM fusion model, the graph develops during the 1500–2000 h period with an entirely different tendency. However, this is also correlated with the periodicity of Informer fusion models. The periodicity with the measured data appears to be stable around 1500 h. The graph of the observation data is parallel to the downward direction of the y axis.

## 6. Conclusions

In this paper, we have introduced an informer-based temperature prediction methodology that incorporates time-periodic information. The results of our experiments demonstrate that the inclusion of time-periodic information enhanced the accuracy of our time series predictions. Although the informer did not perform well in all the forecast time zones, it showed a superior performance in several areas, thereby successfully superseding the LSTM structure for temperature prediction. Our most effective approach involved sending the encoder output from the informer and the feature extraction output from the CNN–BLSTM to the decoder using scaled dot product attention.

This study has demonstrated the effectiveness of the Informer-based model in temperature prediction by fusioning AWS data and LADSP image data. In particular, the Informer encoder fusion and Informer encoder–decoder fusion model yielded lower RMSE and MAE values than either the BLSTM-based or the CNN–BLSTM-based fusion models did. The performance of the Informer encoder fusion model showed the lowest RMSE and MAE values when the prediction intervals were greater than or equal to 24 h, while those of the Informer encoder–decoder fusion model were the best when the prediction times were shorter than 24 h. The performance comparison between the Informer fusion models and the CNN–BLSTM fusion model showed that the Informer encoder–decoder fusion model achieved reductions of the RMSE and MAE of 0.07 °C and 0.05 °C, respectively, at the 6 h prediction time, which were compared with those of the CNN–BLSTM fusion model. Similarly, at the 12 h prediction time, the Informer encoder–decoder fusion model outperformed the CNN–BLSTM fusion model, with RMSE and MAE reductions of 0.47 °C and 0.34 °C, respectively. Although the improvement was less significant at the 24 h prediction time, the Informer encoder–decoder fusion model still demonstrated enhanced temperature prediction capabilities compared with those of the CNN–BLSTM fusion model. Furthermore, when we employed the Informer encoder fusion model for temperature prediction, it exhibited significant improvements by reducing the RMSE and MAE by 0.25 °C and 0.19 °C, respectively, at the 24 h prediction time, and by 0.29 °C and 0.20 °C, respectively, at the 336 h prediction time, which was compared with the CNN–BLSTM fusion model.

For future studies, although our present objective was to predict a single temperature variable, future research must also include multivariate forecasting that predicts all input variables concurrently. Moreover, the development of a model with resilience to all weather variables and with applicability to regions beyond the Korean Peninsula is critical. Current models predominantly reflect local effects, but understanding the impact of large air masses and long-term climatic trends on weather forecasting is an important avenue for future research. Additionally, ongoing work should emphasize the evolution of time series prediction models. In subsequent studies, we plan to incorporate cutting-edge models such as Autoformer [33] and N-hits [40] and explore the development of an innovative time series prediction model.

## Figures and Tables

**Table 1:** Three types of NWP models provided by KMA.

Model	Horizontal Resolution (Vertical Layers)	Number of Variables (Isobaric/Single)	Forecast Period (H)	Forecast Cycle (H)	Number of Prediction per Day	Grid Size (Coordinates)
GDAPS	0.3515625° (70)	7/101	0~84 90~288	3 6	8 4	1024 × 769 (0° E, 90° N)
RDAPS	12 km (70)	9/101	0~87	6	4	491 × 419 (101.577323° E, 12.217029° N)
LDAPS	1.5 km (70)	8/78	36	3	8	602 × 781 (121.834429° E, 32.256875° N)

**Table 2:** Comparison of the root-mean-square error (RMSE) and mean absolute error (MAE) between BLSTM and Informer using only observed data.

Time (H)	Metric	BLSTM	Informer
6	RMSE	2.38	1.62
	MAE	1.75	1.20
12	RMSE	2.70	2.39
	MAE	1.96	1.90
24	RMSE	3.05	2.92
	MAE	2.28	2.26
72	RMSE	3.83	3.27
	MAE	2.94	2.53
168	RMSE	4.08	3.74
	MAE	3.14	2.91
336	RMSE	4.42	4.05
	MAE	3.40	3.15

**Table 3:** Comparison of the root-mean-square error (RMSE) and the mean absolute error (MAE) between various Informer fusion models. The results of the model that most closely predicted the target value observed in data collected by AWS are highlighted in bold; the performance of the CNN–BLSTM fusion model is the same as the best model in [32].

Time (H)	Metric	CNN–BLSTM Fusion Model [32]	Informer Fusion Model			
			Embedding Addition	Encoder	Decoder	Encoder Decoder
6	RMSE	0.92	1.69	0.86	0.97	**0.85**
	MAE	0.72	1.28	0.68	0.78	**0.67**
12	RMSE	1.62	2.62	1.21	1.44	**1.15**
	MAE	1.23	2.07	0.93	1.13	**0.89**
24	RMSE	1.98	2.93	**1.73**	2.15	1.91
	MAE	1.49	2.22	**1.30**	1.60	1.39
72	RMSE	3.14	3.81	**2.99**	3.27	3.22
	MAE	2.42	2.90	**2.27**	2.42	2.41
168	RMSE	3.74	4.50	**3.47**	4.23	4.36
	MAE	2.88	3.58	**2.59**	3.35	3.36
336	RMSE	4.26	4.88	**3.97**	4.97	4.72
	MAE	3.29	3.94	**3.09**	3.95	3.83

## Data Availability

This study used public data provided by KMA. Two types of data were utilized: AWS, which is observed data, and NWP, which includes GDAPS, RDAPS and LDAPS. AWS can be downloaded from https://data.kma.go.kr/data/grnd/selectAwsRltmList.do?pgmNo=56 (accessed on 5 July 2023). NWP can be downloaded from https://data.kma.go.kr/data/rmt/rmtList.do?code=340&pgmNo=65 (accessed on 5 July 2023). Requests for large volumes of these data should be made to KMA.
